# Editorial Notes

**Published:** 1926

**Authors:** 


### Editorial Notes.

**Resignation of Editor.**

It is with feelings of great regret that we record the resignation of Dr. P. Watson-Williams from the editorial chair. Dr. Watson-Williams had completed twenty-five years of service on the Editorial Committee, which he had joined in January, 1900, in the capacity of Assistant Editor. He remained Assistant Editor until December, 1912, when he was appointed Editor in succession to Dr. Shingleton Smith, who had resigned. Thus the chief part of Dr. Watson-Williams' editorship coincided with the war and the post-war years. It was during this period that he rendered what were perhaps his most valuable services to the *Journal* and the Society. In spite of his own pre-occupation with military duties in a consultative capacity in the Southern Command, and in spite of the continued absence of many members of the Editorial Committee, the *Bristol Medico-Chirurgical Journal* did not suspend publication during the war. This was undoubtedly due to the energy and enthusiasm which the Editor displayed, and to his unshaken belief in the destinies of our Country and Empire. But in the post-war period the difficulties were even greater. Costs of publication increased rapidly, and at first the membership of the Society was depleted by war casualties and the lack of newly-qualified practitioners. Nevertheless, Dr. Watson-Williams determined that the difficulties could only be temporary, and he therefore spared nothing to keep the *Journal* alive and add to its interest. His knowledge of the technical side of printing, and more particularly of the art of illustration, enabled him to suggest many improvements, until the issue of January, 1926, seemed to satisfy his aspirations.

Dr. Watson-Williams is himself a good draughtsman, and it is particularly in the quality and quantity of the illustrations in the *Journal* that so great an improvement has been made under his editorship. His own numerous and important contributions to the *Journal* were generally freely illustrated by his own drawings.

He laid down the editorial pen and handed on to his successors the *Journal* in a form which, as he wrote in his farewell editorial notes, "it is believed will enhance its value to our readers," and be "more worthy of the intimate association of the Medico-Chirurgical Society with the University of Bristol."

Dr. Watson-Williams will now be free to devote to his practice those energies which he has so cheerfully given to the Journal, and for which we can only offer him as reward our thanks and praise for work well done.

**Royal Institute of Public Health.**

THE Annual Congress of the Royal Institute of Public Health was held in the University of Bristol from 19th to 25th May.

Among the many interesting papers that were contributed, Dr. D. S. Davies's "Random Recollections of Forty Years' Service" deserves special mention, dealing with the various outbreaks of epidemic disease in Bristol, and the many introductions of epidemic disease which, by the vigilance of the Health Department, did not lead to outbreaks. Dr. Carey Coombs and Dr. Hadfield spoke on the pathology of cardiac infections, and Dr. Macdonald Critchley on "Congenital Word-blindness and Mirror Writing." There were many social entertainments, including evening receptions by the Lord Mayor and Lady Mayoress of Bristol and by the Council of the University.

The Duke of Beaufort invited a party to Badminton, where the hounds and stables were shown to the visitors. There were other excursions to the noted beauty spots in the neighbourhood of Bristol. The local Secretaries, Professor Walker Hall and Dr. R. A. Askins, are to be congratulated on the success attending their efforts on behalf of the Congress.

**University of Strasbourg.**

Two interesting postgraduate courses are announced to be held this year in Strasbourg. The first course begins on October 8th and continues until October 23rd. It is devoted to the study of pulmonary tuberculosis and diseases of the respiratory passages. The fee for the course is 250 francs. Applications should be sent to Dr. Vaucher, 8 Quai Finkwiller, Strasbourg. The second course deals with cancer, and will extend from October 18th to November 6th. The fee is 250 francs. Applications should be sent to Dr. Gunsett, Directeur du Centre Anticancéreux. Hôpital Civil, Strasbourg. Our readers may welcome these opportunities of visiting this old University town and studying under the distinguished professors who are taking part in the courses.

